# Long non-coding RNA MYU promotes ovarian cancer cell proliferation by sponging miR-6827-5p and upregulating HMGA1

**DOI:** 10.3389/pore.2023.1610870

**Published:** 2023-01-27

**Authors:** Shaoyu Wang, Qiaomei Zheng, Jinhua Wang, Shaozhan Chen, Lihong Chen

**Affiliations:** ^1^ Department of Obstetrics and Gynecology, The First Affiliated Hospital, Fujian Medical University, Fuzhou, China; ^2^ Department of Obstetrics and Gynecology, National Regional Medical Center, Binhai Campus of the First Affiliated Hospital, Fujian Medical University, Fuzhou, China; ^3^ Fujian Key Laboratory of Precision Medicine for Cancer, The First Affiliated Hospital, Fujian Medical University, Fuzhou, China

**Keywords:** lncRNA, ovarian cancer, HMGA1, MYU, miR-6827-5p

## Abstract

**Background:** Long non-coding RNAs (lncRNAs) have been confirmed to play vital roles in tumorigenesis. LncRNA MYU has recently been reported as an oncogene in several kinds of tumors. However, MYU’s expression status and potential involvement in ovarian cancer (OC) remain unclear. In this study, we explored the underlying role of MYU in OC.

**Methods and results:** The expression of MYU was upregulated in OC tissues, and MYU’s overexpression was significantly correlated with the FIGO stage and lymphatic metastasis. Knockdown of MYU inhibited cell proliferation in SKOV3 and A2780 cells. Mechanistically, MYU directly interacted with miR-6827-5p in OC cells; HMGA1 is a downstream target gene of miR-6827-5p. Furthermore, MYU knockdown increased the expression of miR-6827-5p and decreased the expression of HMGA1. Restoration of HMGA1 expression reversed the influence on cell proliferation caused by MYU knockdown.

**Conclusion:** MYU functions as a ceRNA that positively regulates HMGA1 expression by sponging miR-6827-5p in OC cells, which may provide a potential target and biomarker for the diagnosis or prognosis of OC.

## Introduction

Ovarian cancer (OC) is a common gynecological malignant tumor with the highest mortality rate. There will be approximately 57,090 newly diagnosed ovarian cancer cases and 39,306 deaths in China in 2022. Due to the insidious onset, there is no obvious clinical symptom at the early stage, 70% of patients are diagnosed at the advanced stage. Despite the improvements in surgery, chemotherapy, and maintenance therapy, the 5-year survival rate is still low, only about 47%. The main causes of death are recurrence and distant metastasis. Therefore, there is an urgent need to further investigate the molecular mechanisms and find novel therapeutic targets in ovarian cancer (1-3).

The Human Genome Project has shown that only 1%–2% of nucleic acid sequences encode proteins. The rest without the ability to encode protein includes long non-coding RNAs (LncRNAs) and short RNAs represented by microRNA (miRNA) and siRNA (4). Numerous studies have confirmed that ncRNAs play critical roles in tumorigenesis and cancer progression. LncRNAs are a group of transcripts with a length of more than 200 nucleotides (nt) (5). LncRNAs can exert a tumor-promoting or tumor-suppressing effect in different cancers. MiRNAs are another endogenous short non-coding RNA with a length of approximately 17-25 nt. MiRNAs bind to the 3′-untranslated region (UTR) of target mRNA, leading to mRNA degradation or translation inhibition (6). Competitive endogenous RNA (ceRNA) is a typical post-transcriptional mechanism in which lncRNAs competitively sponge certain miRNAs and then positively regulate downstream mRNAs (7).

The c-Myc upregulated lncRNA MYU was named by Yoshihiro in 2016 (8). MYU is also annotated as VPS9D1-AS1 because it generates from the opposite strand of the *VPS9D1* gene, which is located in human chromosome 16 q24.3. It has been reported in several common types of human cancers in recent years. Several studies (8-14) have confirmed its critical role in the biological process of tumors, including cell proliferation, apoptosis, invasion, and migration. For instance, MYU interacted with RNA binding protein hnRNP-K to upregulate the CDK6 expression in colon cancer (8). In prostate cancer (PCa), MYU promoted tumorigenesis and progression by mediating the miR-184/c-Myc axis (9). Other researchers found it accelerated the cancer progression by sponging miR-4739 to upregulate MEF2D in PCa (10). In non-small cell lung cancer (NSCLC), the overexpression of MYU exhibited a robust correlation with the poor prognosis (11). The mechanism research revealed that MYU acted as a ceRNA to bind to miR-532-3p and upregulate the expression of HMGA2 in NSCLC (12). In acute lymphoblastic leukemia (ALL), MYU promoted cell proliferation by elevating GPX1 expression by sequestering miR-491-5p and miR-214-3p (13). MYU can also sponge miR-491-5p to upregulate SEC61A1 in hepatocellular carcinoma (14). However, to date, there have been no reports on the role or mechanism of MYU in OC.

The present study is the first to investigate the expression of MYU in OC tissue samples, and to observe the correlation between MYU expression and clinicopathologic features. Furthermore, we focused on the effects of MYU on cancer cell proliferation *in vitro*. These results will help us to clarify the role and potential involvement mechanism of MYU in OC.

## Materials and methods

### Sample collection

20 epithelial ovarian cancer tissue samples and 20 controls were obtained from patients who underwent surgical resection at the Department of Obstetrics and Gynecology, the First Affiliated Hospital of Fujian Medical University (Fujian, China) between March 2021 and January 2022. Normal ovarian tissue specimens were obtained from patients who underwent hysterectomy and bilateral salpingo-oophorectomy due to benign uterine diseases, including leiomyoma, adenomyosis, or uterine prolapse. The patients with ovarian cancer did not receive preoperative chemotherapy or have any concomitant gynecological cancer. Their clinical information is summarized in Table 1. All resected tissue samples were stored at −80°C after adding RNA Stabilization Reagent (Beyotime, Shanghai, China). This study was approved by the Ethics Committee of First Affiliated Hospital of Fujian Medical University (No. [2020]398). Informed consent was obtained from all patients included in the study.

**TABLE 1:** Relationship between MYU expression and clinicopathologic parameters of OC patients.

Characteristics	Number of cases	MYU expression		*p*-Value
		High (*n* = 10)	Low (*n* = 10)	
age (years)				0.07
≤55	9	2	7	
>55	11	8	3	
Histological type				0.303
Serous	15	9	6	
Others	5	1	4	
FIGO stage				0.005
I-II	11	2	9	
III-IV	9	8	1	
Lymph node metastasis				0.02
Positive	8	7	1	
Negative	12	3	9	

### Cell culture

Human ovarian cancer cell lines (SKOV3 and A2780) were obtained from the Fujian Key Laboratory of Precision Medicine for Cancer. The cells were cultured in RPMI-1640 medium supplemented with 10% fetal bovine serum (FBS) and 1% penicillin-streptomycin. The medium was renewed every 2 or 3 days. All cells were maintained in a humidified incubator containing 5% CO_2_ at 37°C.

### RNA extraction and quantitative real-time reverse-transcription polymerase chain reaction (qRT-PCR)

Trizol reagent (Beyotime, Shanghai, China) was used to extract total RNA from tissue samples and cell culture. The RNA concentration was determined by an ND5000 spectrophotometer (BioTeke, Beijing, China). RNA (1ug) was reverse transcribed to cDNA using a PrimeScript™ RT Master Mix (TaKaRa, Japan). Quantitative real-time polymerase chain reaction (PCR) was performed on 7500 Real-Time PCR systems (Applied Biosystems Life Technologies, United States). TB Green® Fast qPCR Mix (TaKaRa Bio, Otsu, Japan)was used to determine the mRNA expression, with GAPDH as control. For miRNA, RNAiso (TaKaRa, Japan) was used to extract miRNA and the Small RNA Cloning Kit (TaKaRa, Japan) was used for reverse transcription. Then Mir-X miRNA qRT-PCR TB Green® Kit (TaKaRa, Japan) was used to determine the expression of miRNA, with U6 as control. The relative expression of RNA was analyzed by the 2^−ΔΔCT^ method. The primer sequences used for qRT-PCR were listed in Table 2.

**TABLE 2:** Primer sequences used for RT-qPCR.

Gene	Primer sequences (5′–3′)	
MYU	Forward	AGT​GGC​CGT​TTT​ACA​GAG​ACA
	Reverse	CAT​GCC​AAG​CTA​CGG​GAA​GG
HMGA1	Forward	AAGGGGCAGACCCAAAAA
	Reverse	TCC​AGT​CCC​AGA​AGG​AAG​C
GAPDH	Forward	GGA​GCG​AGA​TCC​CTC​CAA​AAT
	Reverse	GGC​TGT​TGT​CAT​ACT​TCT​CAT​GG
miR-6827-5p	Forward	AAG​CAA​CCC​TTA​ACT​CCA​GA
	Reverse	CCA​AGC​AAC​CCT​TAA​CTC​CA
U6	Forward	CTCGCTTCGGCAGCACA
	Reverse	AAC​GCT​TCA​CGA​ATT​TGC​GT

### shRNA transfection

The short-hairpin RNA (shRNA) lentiviruses targeting MYU and control shRNA lentiviruses were inserted into the pHBLV-CMV-MCS-3FLAG-EF1-ZsGreen-T2A-PURO vector (Hanbio Biotechnology, Shanghai, China). The combined plasmids were named sh-MYU-1#/2#/3#. The shRNA lentiviruses targeting miR-6827-5p were inserted into the pHBLV-CMV-ZsGreen-T2A-Puro vector (Hanbio Biotechnology, Shanghai, China). The combined plasmids were named miR-6827-5p inhibitor-1#/2#/3#. The sequences were shown in Table 3. The SKOV3 and A2780 cells were transfected with plasmids using PolyJet^TM^ *In Vitro* DNA Transfection Reagent (SignaGen, MD, United States) according to the reagent protocol. Cells that were transfected with empty plasmids (sh-NC and NC inhibitor) served as controls. Transfected cells were obtained 2 days later and transfection efficiency was evaluated by qRT-PCR.

**TABLE 3:** The sequences of interference vectors.

Name	Sequences (5′–3′)	
sh-MYU-1#	Top strand	GAT​CCG​ACC​ATG​GAC​CTG​CTC​ACA​CAG​CCC​TTC​TTT​CAA​GAG
		AAG​AAG​GGC​TGT​GTG​AGC​AGG​TCC​ATG​GTC​TTT​TTT​G
	Bottom strand	AAT​TCA​AAA​AAG​ACC​ATG​GAC​CTG​CTC​ACA​CAG​CCC​TTC​TTC
		TCT​TGA​AAG​AAG​GGC​TGT​GTG​AGC​AGG​TCC​ATG​GTC​G
sh-MYU-2#	Top strand	AGA​CCC​CAG​GAA​GCC​CAG​TGG​TGT​CTG​GAC​ACC​AGA​GGA​GT
		CTC​TCT​CAT​CCT​CCC​GGA​TTG​CTC​TGA GG CCAGGAGC
	Bottom strand	GCC​TTC​CCG​TAG​CTT​GGC​ATG​GAG​CAC​CTC​TGC​GCG​ACC​ATG
		GAC​CTG​CTC​ACA​CAG​CCC​TTC​TGC​GC GCCGTGGGAGC
sh-MYU-3#	Top strand	GAT​CCG​CCA​CTG​GAG​TTC​CTC​TGT​CTT​CTG​GGA​CTT​CAA​GAG
		AGT​CCC​AGA​AGA​CAG​AGG​AAC​TCC​AGT​GGC​TTT​TTT​G
	Bottom strand	AAT​TCA​AAA​AAG​CCA​CTG​GAG​TTC​CTC​TGT​CTT​CTG​GGA​CTC
		TCT​TGA​AGT​CCC​AGA​AGA​CAG​AGG​AAC​TCC​AGT​GGC​G
miR-6827-5p inhibitor1#		ACC​CTC​GGT​TAT​TCC​AGA​CAC​G
miR-6827-5p inhibitor2#		ACC​CTC​GGT​ACT​AAT​AAA​CAC​G
miR-6827-5p inhibitor3#		ACC​CTC​GGT​ACT​CCG​TGA​TAC​G

### CCK-8 assay

CCK8 assay was performed to detect cell proliferation. Transfected SKOV3 and A2780 cells were seeded into 96-well plates (2 × 10^3^ cells and 100ul each well) at 37°C for 24 h, 48 h, or 72 h. Then the cells were incubated with 10 μL Cell Counting Kit-8 (Fudebio, Hangzhou, China) solution for 4 h. The absorbance at 450 nm was determined by utilizing a SpectraMax iD3 multi-function reader (Molecular Devices, United States).

### Dual-luciferase reporter assay

The sequence of miR-6827-5p was inserted into the PGL3-CMV-LUC-MCS vector (miR-6827-5p WT). The 5′UTR sequence of miR-6827-5p which contained the binding site of MYU was mutated and inserted into the same luciferase reporter vector (miR-6827-5p Mut). The recombinant plasmids were synthesized by Genomeditech (Shanghai, China). SKOV3 and A2780 cells were seeded in 24-well plates one night before the transfection. The miR-6827-5p WT or Mut recombinant plasmids and sh-MYU or sh-NC were co-transfected into SKOV3 and A2780 cells using a PolyJet *In Vitro* DNA Transfection Reagent (SignaGen, MD, United States). After 48 h transfection, the cells were collected, and then the luciferase activities were measured using a Dual-Luciferase Reporter Assay (Fudebio, Hangzhou, China). The activity of the firefly luciferase was normalized to that of the renilla luciferase.

Similarly, the 3′UTR sequence of HMGA1 which contained miR-6827-5p binding sites was inserted into the PGL3-CMV-LUC-MCS vector (HMGA1 WT). The 3′UTR sequence of HMGA1 which interacted with miR-6827-5p was mutated and inserted into the equivalent vector (HMGA1 Mut). The HMGA1 WT or Mut recombinant plasmids and miR-6827-5p inhibitor or NC inhibitor were co-transfected into SKOV3 and A2780 cells. The luciferase activities were measured in the same way.

### Western blotting

Total protein was extracted from cells using a Protein Extraction Kit (Solarbio, Beijing, China). The concentration of protein was determined using a BCA Protein Assay Kit (Solarbio, Beijing, China) according to the manufacturer’s instructions. Then protein samples (40ug) were separated on sodium dodecyl sulfate 10% polyacrylamide gel electrophoresis (SDS-PAGE, Fudebio, Hangzhou, China) and transferred onto polyvinylidene difluoride (PVDF) membrane (Thermo Scientific, MA, United States). After being incubated with 5% BSA for 1 h at room temperature, the membranes were incubated with primary antibodies targeting HMGA1 (29895-1-AP; dilution 1:10000; Proteintech Group, United States) or GAPDH (#5174; dilution 1:1000; Cell Signaling Technology, United States) at 4°C overnight. After Tris-buffered saline containing 0.1% Tween-20 (TBST) was washed three times, membranes were incubated with HRP-linked goat anti-rabbit secondary antibody (#7074; dilution 1:1000; Cell Signaling Technology, United States) at room temperature for 1 h. Protein bands were visualized using Pierce™ ECL Western Blotting Substrate (Thermo Scientific, MA, United States) with chemiluminescence detection system (ChemiDoc, Bio-Rad, United States). GAPDH was used as the loading control for relative protein expression.

### Statistical analysis

All experiments were independently performed in triplicate with 3 repeats. SPSS 26(Chicago, IL, United States) or GraphPad Prism 9.0 software (GraphPad, San Diego, CA, United States) was used for data processing and analysis. Data were presented as mean ± standard deviation (SD). Pairwise comparisons were performed using Student’s t-test. The differences among multiple groups (≥3) were carried out using one-way analysis of variance (ANOVA), followed by the *post hoc* Tukey’s test, if appropriate. The correlation between MYU and clinicopathologic parameters of OC patients was analyzed using Fisher’s exact tests. *p*-value < 0.05 was considered statistically significant.

## Results

### The expression of MYU in ovarian cancer tissues and its relationship with clinical significance

**FIGURE 1:**
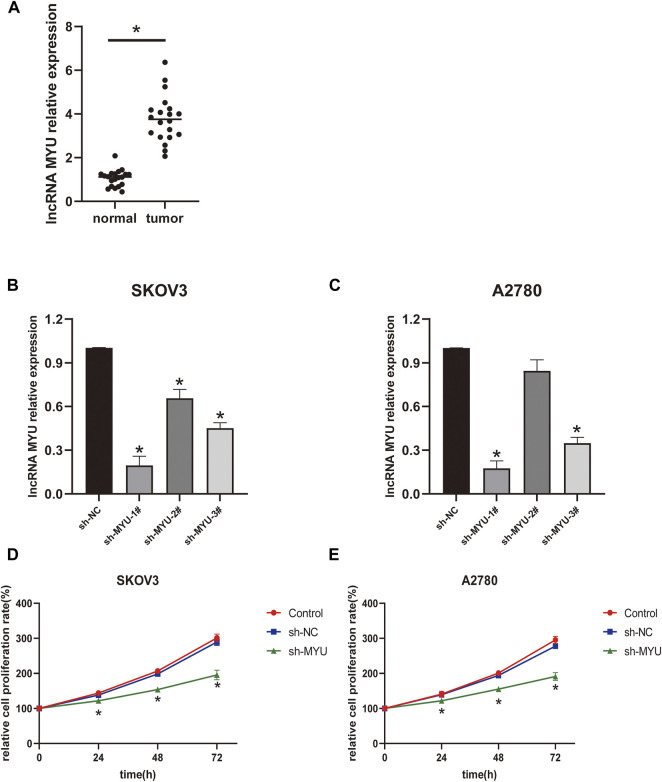
Relative expression of MYU in OC tissues and the effect of MYU on the proliferation of OC cell lines. **(A)** The expression level of MYU in OC tissues was higher than that in normal ovarian tissues by qRT-PCR. *p < 0.05, compared with normal tissue group. **(B, C)** The silencing efficiency of sh-MYU in SKOV3 and A2780 cells were detected by qRT-PCR; **(D, E)** Knockdown of MYU inhibited OC cell proliferation. *p < 0.05, compared with the sh-NC.

QRT-PCR was used to detect the expression of MYU in OC tissues and normal ovarian tissues. The expression of MYU was significantly higher in OC tissues than in normal tissues (Figure 1A). To further examine the significance of MYU expression in ovarian cancer, MYU expression was correlated with various clinicopathological features. Taking the median of MYU expression in OC tissues as the cut-off value, 20 OC patients were divided into MYU high expression group (*n* = 10) and MYU low expression group (*n* = 10) for clinicopathological correlation analysis. As shown in Table 1, the overexpression of MYU in OC was significantly correlated with the FIGO stage and lymphatic metastasis but was not correlated with patient age and histological type.

### MYU silencing inhibits ovarian cancer cell proliferation

The transfection efficiency of MYU was shown in Figures 1B, C; In SKOV3 cells, the silencing efficiency of the three types of sh-MYU was significantly higher than that of sh-NC. Sh-MYU-1# had the best silencing efficiency. In A2780 cells, the silencing efficiency of sh-MYU-1# and sh-MYU-3# were significant, and sh-MYU-1# was better. As a result, sh-MYU-1# was selected for further experiments. The results of the CCK8 assay revealed that MYU silencing significantly inhibited the viability and proliferation of SKOV3 and A2780 cells (Figures 1D, E).

### MYU binds with miR-6827-5p and MYU silencing promotes its expression

**FIGURE 2:**
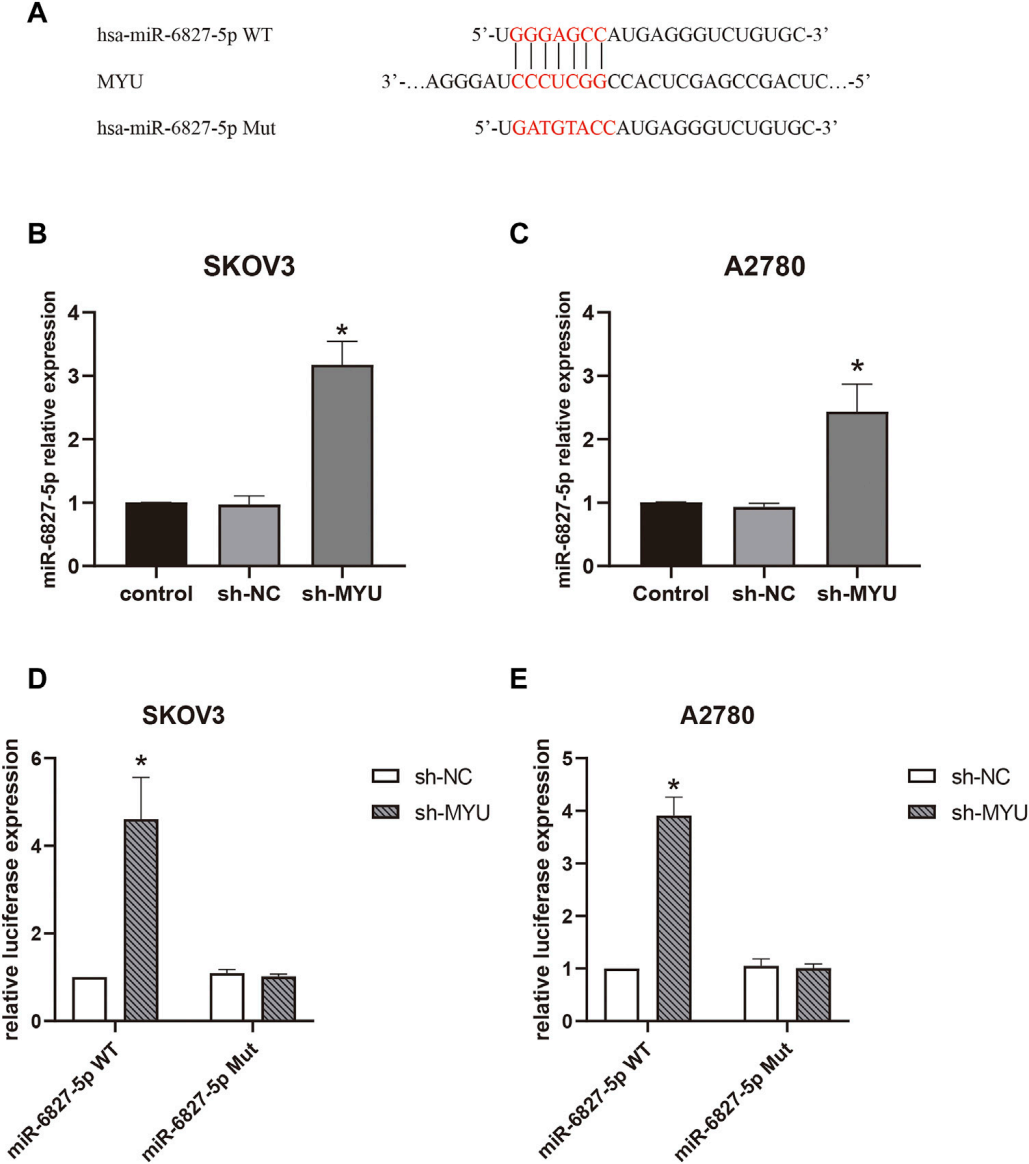
MYU functions as a sponge for miR-6827-5p in OC cells. **(A)** Wild-type and mutant MYU binding site in miR-6827-5p. **(B, C)** The relative expression of miR-6527-5p in SKOV3 and A2780 cells with MYU knockdown by qRT-PCR. **(D, E)** Interaction between MYU and miR-6827-5p in OC cells revealed by the luciferase reporter assay. SKOV3 and A2780 cells were co-transfected with a luciferase reporter plasmid carrying miR-6827-5p WT or Mut and sh-MYU or sh-NC. *p < 0.05, compared with the sh-NC.

Then we further investigated the molecular mechanism of MYU in ovarian cancer. Previous studies have revealed that MYU is mostly localized in the cytoplasm of cancer cells (10, 12-14), implying that it may function as a ceRNA by binding to specific miRNAs. To confirm this hypothesis, the bioinformatics tool miRDB (http://mirdb.org/) was used to predict the potential miRNA targets of MYU. There were 42 predicted miRNAs targeting MYU (Supplementary Table S1). The miRNA with the highest target score was miR-6827-5p in the list, so it was chosen for our study. We found that miR-6827-5p had a potential binding sequence of MYU and MYU had 10 potential seed locations of miR-6827-5p. The potential binding sequence was shown in Figure 2A. Compared with sh-NC in qRT-PCR, knockdown of MYU dramatically increased the expression of miR-6827-5p (Figures 2B, C), indicating that MYU negatively regulated miR-6827-5p. Additionally, A luciferase reporter assay was performed. The luciferase activity was significantly increased in miR-6827-5p WT and sh-MYU co-transfected cells, compared with miR-6827-5p WT and sh-NC co-transfected cells. In contrast, the luciferase activity had no significant difference in miR-6827-5p Mut transfected cells (Figures 2D,E). The results revealed that MYU silencing promoted the expression of miR-6827-5p WT in SKOV3 and A2780 cells, while the regulatory effect of MYU disappeared when miR-6827-5p was mutated.

### HMGA1 is a target gene of miR-6827-5p

**FIGURE 3:**
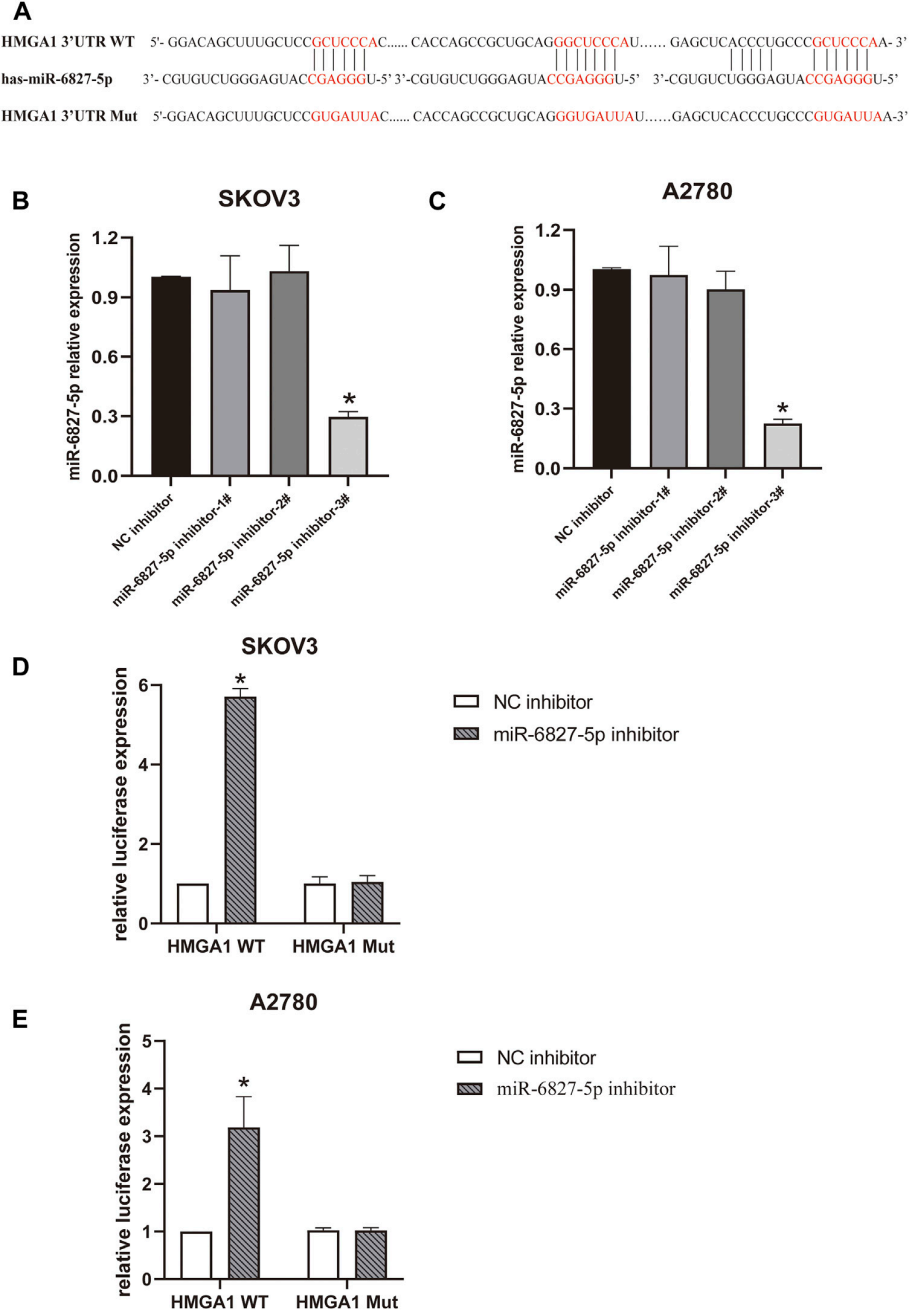
miR-6827-5p targets HMGA1 mRNA. **(A)** Wild-type and mutant miR-6827-5p binding site in the 3′-UTR of HMGA1 mRNA. **(B, C)** The silencing efficiency of miR-6827-5p inhibitor in SKOV3 and A2780 cells were detected by qRT-PCR. **(D, E)** Interaction between HMGA1 mRNA and miR-6827-5p in OC cells revealed by the luciferase reporter assay. SKOV3 and A2780 cells were co-transfected with a luciferase reporter plasmid carrying HMGA1 WT or Mut and miR-6827-5p inhibitor or NC inhibitor. *p < 0.05, compared with the NC inhibitor.

Next, we wanted to explore the downstream target genes of miR-6827-5p in OC. By utilizing targetscan (https://www.targetscan.org/), 3944 transcripts containing a total of 5419 binding sites were predicted. HMGA1 was selected for further investigation because it was reported to be overexpressed in OC and correlated with cancer progression (15-17). We found that there were four potential binding sequences of miR-6827-5p in the 3′-UTR of HMGA1 mRNA. Three of the binding sequences of miR-6827-5p and HMGA1 were shown in Figure 3A. Firstly, the transfection efficiency of miR-6827-5p inhibitor was detected, and the silencing efficiency of miR-6827-5p inhibitor-3# was the best in SKOV3 and A2780 cells, so it was selected for further experiments (Figures 3B, C).

Then, a luciferase reporter assay was used to verify the binding of miR-6827-5p to 3′-UTR of HMGA1 mRNA. The results showed that luciferase activity was significantly increased in miR-6827-5p inhibitor and HMGA1 WT co-transfected cells, compared with NC inhibitor and HMGA1 WT co-transfected cells. In contrast, no evident changes were observed in cells co-transfected with HMGA1 Mut and miR-6827-5p inhibitor or NC inhibitor (Figures 3D, E).

### MYU Positively Regulates HMGA1 Expression *via* Sponging miR-6827-5p

**FIGURE 4:**
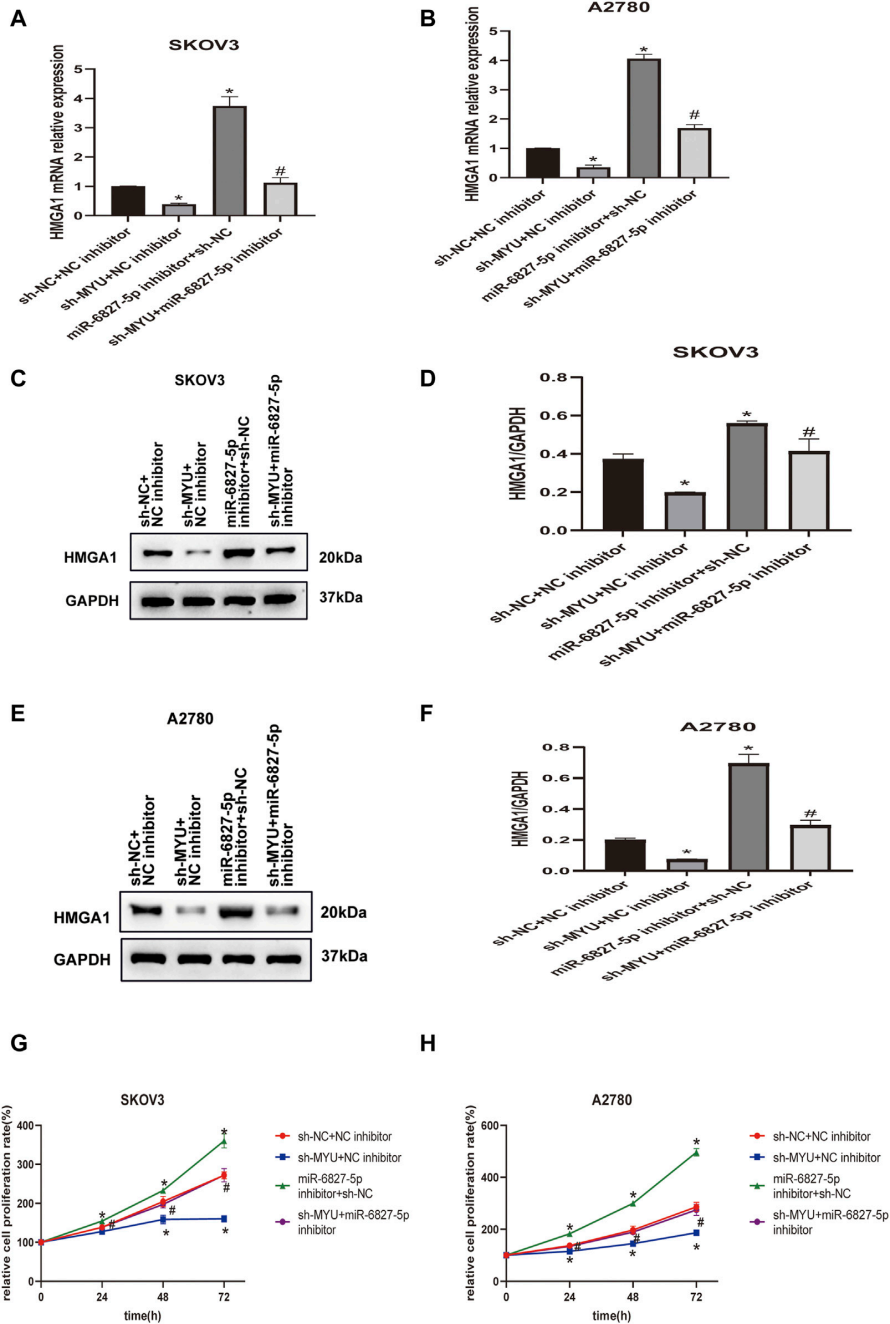
MYU acts as a ceRNA of miR-6827-5p and hence upregulates HMGA1 expression. **(A, B)** HMGA1 mRNA levels in SKOV3 and A2780 cells were determined by qRT-PCR after co-transfecting with sh-NC+ NC inhibitor, sh-MYU + NC inhibitor, miR-6827-5p inhibitor +sh-NC or sh-MYU + miR-6827-5p inhibitor. **(C–F)** HMGA1 protein levels in SKOV3 and A2780 cells were determined by western blot after co-transfecting with sh-NC+ NC inhibitor, sh-MYU + NC inhibitor, miR-6827-5p inhibitor +sh-NC or sh-MYU + miR-6827-5p inhibitor. **(G, H)** co-transfecting with sh-MYU and miR-6827-5p inhibitor reversed the influence on cell proliferation caused by MYU knockdown in SKOV3 and A2780 cells. *p < 0.05, compared with the sh-NC + NC inhibitor. ^#^ *p* < 0.05, compared with the sh- MYU + NC inhibitor or miR-6827-5p inhibitor +sh-NC.

To further investigate whether MYU could modulate HMGA1 expression by sponging miR-6827-5p, rescue assays were carried out. As revealed by qRT-PCR(Figures 4A, B) and western blot (Figures 4C–F), the expression of HMGA1 mRNA and protein were decreased in SKOV3 and A2780 cells when MYU silencing, and the expression of HMGA1 mRNA and protein were remarkably increased when miR-6827-5p silencing. However, when co-transfecting with sh-MYU and miR-6827-5p inhibitor, the influence of MYU knockdown on HMGA1 mRNA and protein expression was abrogated. CCK-8 assay showed that knockdown of miR-6827-5p promoted OC cell proliferation. However, there was no significant change in cell proliferation after co-transfecting with sh-MYU and miR-6827-5p inhibitor. The results indicated that the restoration of HMGA1 expression reversed the effect of MYU knockdown on cell proliferation (Figures 4G, H). In other words, MYU sponged miR-6827-5p, thereby enhancing HMGA1 expression and promoting cell proliferation in OC.

## Discussion

A growing number of studies have revealed that the aberrant expression of lncRNAs is closely related to the occurrence and development of tumors. LncRNAs are expected to become molecular markers and therapeutic targets for tumor diagnosis and prognosis (18). The function of several lncRNAs has been revealed in ovarian cancer, such as MALAT1 (19), HOTAIR (20), CCAT1 (21)、NEAT1 (22), MNX1-AS1 (23).

Although the oncogenic role of MYU in a variety of malignant tumors has been gradually discovered in recent years (8-14), the expression status and potential involvement of MYU in ovarian cancer remain unclear. In the present study, we investigated the expression of MYU in OC tissues and analyzed the clinical significance of MYU in OC. It is optimal to obtain tumor tissues and adjacent non-tumor tissues for evaluation from the same patients. However, in many cases, the ovarian tumor was large and the resected tissue samples should be stored at −80°C immediately. It is difficult to ensure that there is no infiltration of tumor cells if we collect the tissue adjacent to the tumor. Just like other studies (24, 25), we collected OC and normal ovarian tissue samples from different patients. We found that the MYU expression level was higher in OC tissues than that in normal ovarian tissues. The expression of MYU was positively associated with the FIGO stage and lymphatic metastasis. Moreover, shRNA-mediated knockdown of MYU significantly inhibited OC cell proliferation. Therefore, MYU exerts a tumor-promoting effect in OC and it might be a potential prognostic biomarker in OC patients.

Furthermore, we explored the molecular mechanism by which MYU contributed to tumorigenesis. In the recent decade, the ceRNA network has been extensively studied in the field of oncology (7). LncRNA can function as a ceRNA by competitively sponging miRNAs to release mRNAs from RNA-induced silencing complexes (RISCs). The bioinformatics tool was used to predict the potential miRNAs that interacted with MYU.

In the present study, miR-6827-5p expression was at a higher level after MYU knockdown compared with sh-NC in OC cells. It was also disclosed that the expression of miR-6827-5p was negatively regulated by MYU. Luciferase reporter assay verified the combination between MYU and miR-6827-5p. Function test showed that knockdown of miR-6827-5p promoted cell proliferation. These results displayed that MYU promoted cell proliferation by sponging miR-6827-5p in OC.

Based on the bioinformatics website’s predicted results, we found HMGA1 was a target gene of miR-6827-5p. HMGA1 is a member of the high mobility group (HMG) protein family and includes three isoforms: HMGA1a (107 amino acids, 11.7 kDa), HMGA1b (96 amino acids, 10.6 kDa) and HMGA1c (179 amino acids, 19.6 kDa) isoforms, which result from translation of alternative spliced forms of The *HMGA1* gene (26). HMGA1a and HMGA1b are identical in sequence except for the absence of a 11 amino acid region. HMGA1c has in common with HMGA1a only the first 64 amino acid, because of the alternative splicing using non-canonical splice donor and acceptor sites (27). HMGA1 protein binds to the A/T-rich DNA sequences and shows antagonistic actions against histone H1, leading to an open chromatin structure and promoting transcription (28).

HMGA1 is overexpressed in most cancers, including OC (15). The overexpression of HMGA1 is related to the malignant degree of the tumor, and knockdown of HMGA1 can reduce the proliferation and invasion ability of OC cells *in vitro* (17). In the present study, both the mRNA and protein expression of HMGA1 decreased when MYU was silenced, and there was a positive correlation between MYU and HMGA1 expression. These results suggested that MYU regulated HMGA1 expression in a post-transcriptional manner. Meanwhile, the mRNA and protein expression of HMGA1 increased when miR-6827-5p was silenced in OC cells. Luciferase reporter assay verified the interaction between miR-6827-5p and HMGA1 3′-UTR. The results of rescue experiments demonstrated that the effects of MYU knockdown on cell proliferation could be reversed by the upregulation of HMGA1. There was only one band of HMGA1 that could be observed in western blotting, and the molecular weight was about 20 KDa. Given the difference in molecular weight between HMGA1a, HMGA1b and HMGA1c, we speculated that it might be HMGA1c that regulated by the MYU/miR-6827-5p axis. In future studies, we will further explore the role of different HMGA1 protein isoforms on ovarian cancer.

Our study revealed that MYU was upregulated in ovarian cancer and it functioned as an oncogene, because knockdown of MYU can inhibit cell proliferation. However, there are limitations in our current study. First, whether MYU can promote OC cell migration and invasion remains to be explored. Second, the results of this study are limited to *in vitro* studies, tumor xenograft experiments are urgently needed. Third, lncRNA can regulate the expression of genes by sponging multiple different miRNAs and controlling transcriptional and post-transcriptional regulation by binding with proteins. A miRNA can regulate the expression of multiple target genes. Therefore, MYU/miR-6827-5p/HMGA1 is likely to be only one of the possible pathways in tumorigenesis of OC. We need to conduct more in-depth studies in the future.

## Conclusion

In this study, we first elucidated that the aberrant overexpression of MYU promoted ovarian cancer cell proliferation by interacting with miR-6827-5p and upregulating HMGA1 expression. The novel ceRNA network composed of MYU, miR-6827-5p, and HMGA1 may provide potential targets and biomarkers for the diagnosis and prognosis of ovarian cancer.

## Data Availability

The raw data supporting the conclusion of this article will be made available by the authors, without undue reservation.
