# Supplementary material for: Long non-coding RNA MYU promotes ovarian cancer cell proliferation by sponging miR-6827-5p and upregulating HMGA1
Source: Pathol Oncol Res. 2023 Jan 27;29:1610870. doi: 10.3389/pore.2023.1610870 (PMC9911462; doi:10.3389/pore.2023.1610870)
Supplement: Supplementary file 1 [file Table1.DOCX]

**Supplementary Table 1** the information of miRNAs predicted by miRDB

| target rank | target score | miRNA name |
| --- | --- | --- |
| 1 | 92 | hsa-miR-6827-5p |
| 2 | 86 | hsa-miR-4252 |
| 3 | 85 | hsa-miR-6870-5p |
| 4 | 84 | hsa-miR-7111-5p |
| 5 | 84 | hsa-miR-4257 |
| 6 | 83 | hsa-miR-6798-5p |
| 7 | 82 | hsa-miR-5698 |
| 8 | 82 | hsa-miR-4723-5p |
| 9 | 81 | hsa-miR-4689 |
| 10 | 80 | hsa-miR-939-5p |
| 11 | 80 | hsa-miR-1343-5p |
| 12 | 73 | hsa-miR-505-5p |
| 13 | 72 | hsa-miR-6778-5p |
| 14 | 72 | hsa-miR-1233-5p |
| 15 | 71 | hsa-miR-3654 |
| 16 | 66 | hsa-miR-6852-5p |
| 17 | 66 | hsa-miR-331-3p |
| 18 | 65 | hsa-miR-6858-5p |
| 19 | 64 | hsa-miR-6794-5p |
| 20 | 63 | hsa-miR-4716-3p |
| 21 | 61 | hsa-miR-593-3p |
| 22 | 61 | hsa-miR-6810-5p |
| 23 | 59 | hsa-miR-6745 |
| 24 | 59 | hsa-miR-4648 |
| 25 | 58 | hsa-miR-4787-5p |
| 26 | 57 | hsa-miR-4654 |
| 27 | 57 | hsa-miR-6894-5p |
| 28 | 56 | hsa-miR-6791-5p |
| 29 | 56 | hsa-miR-4292 |
| 30 | 56 | hsa-miR-5695 |
| 31 | 56 | hsa-miR-361-3p |
| 32 | 56 | hsa-miR-525-5p |
| 33 | 56 | hsa-miR-520a-5p |
| 34 | 55 | hsa-miR-1976 |
| 35 | 54 | hsa-miR-6816-5p |
| 36 | 54 | hsa-miR-769-3p |
| 37 | 54 | hsa-miR-450b-3p |
| 38 | 54 | hsa-miR-4769-5p |
| 39 | 53 | hsa-miR-766-5p |
| 40 | 53 | hsa-miR-6736-3p |
| 41 | 52 | hsa-miR-2355-5p |
| 42 | 50 | hsa-miR-92a-2-5p |
